# Editorial: Nitric Oxide in Plants

**DOI:** 10.3389/fpls.2021.705157

**Published:** 2021-06-25

**Authors:** Raquel Valderrama, Mounira Chaki, Juan Carlos Begara-Morales, Marek Petrivalský, Juan B. Barroso

**Affiliations:** ^1^Group of Biochemistry and Cell Signalling in Nitric Oxide, Department of Experimental Biology, Center for Advanced Studies in Olive Grove and Olive Oils, Faculty of Experimental Sciences, University of Jaén, Jaén, Spain; ^2^Department of Biochemistry, Faculty of Science, Palacky University in Olomouc, Olomouc, Czechia

**Keywords:** nitric oxide signaling, phytohormone signaling, plant growth, seed development, fruit ripening, rhizobium-legume symbiosis, nitro-fatty acids, abiotic stress

Nitric oxide (NO) is a multifunctional gaseous signaling molecule implicated in both physiological and pathological functions in plant systems. Since the first publication accounting for the presence of NO in plants in 1979, numerous milestone discoveries have revealed the complexity of its metabolic and signaling networks. This Research Topic on *Nitric Oxide in Plants* comprises 12 manuscripts and aims to provide new insights into the molecular networks of NO through both original articles and detailed reviews related to seed and plant development, abiotic stress responses, signaling, and beneficial plant-microbial interactions.

In the scope of plant development, the role of NO as a root growth regulator is investigated by Oláh et al. in an original article where the interplay between NO and strigolactones (SLs) in stress-free Arabidopsis plants is studied. Authors correlate the deficiency in SL synthesis or signaling with decreased *S*-nitrosoglutathione (GSNO) reductase (GSNOR) protein abundance and activity and conclude underlining the need for functional GSNOR to control NO/*S*-nitrosothiol levels during SL-induced primary root elongation. However, the authors specify that both SL and karrikin phytohormone signaling could have overlapping roles in their experimental design. Another big challenge for plant biotechnology is the improvement of crop productivity. It is well-established the positive correlation between the supply of nitrogen (*N*) fertilizers and the seed yield. In this context, the original article of Nejamkin et al. investigates the link between N availability and NO in the promotion of growth and seed production in transgenic tobacco plants transformed with the *Ostreococcus tauri* NOS gene (*OtNOS*). They show a severe attenuation in the *OtNOS*-promoted stimulation of growth and production in transgenic plants under conditions of nitrogen scarcity and emphasize the beneficial effects of the application of nitrate (${\text{NO}}_{3}^{-}$)-containing fertilizers. The same kind of fertilizers is also recommended by Li et al. in their original article about Lycium fruits development and ripening in order to increase the endogenous NO content. They induced the silencing of the nitrate reductase (NR) gene in fresh fruits to demonstrate that NR-derived NO negatively regulated fruit coloration/ripening by suppressing anthocyanin *de novo* biosynthesis, as well as by redirecting the flavonoid biosynthetic pathway to proanthocyanidins production, a colorless taste factor. They also found the antagonistic effects of NO and abscisic acid (ABA) in the regulation of the coloration of Lycium fruits.

Two manuscripts go in-depth in the study of the role of NO in the nitrogen fixation by the symbiotic interaction between legume and rhizobium partners. Signorelli et al. reviews the current knowledge about the sources and fundamental roles of NO during the different stages of the interaction and discuss the connections between the metabolism of NO and cytokinins, auxin, and ABA signaling pathways. They also report dose- and time-dependent effects of NO on bacterial nitrogenase expression and activity in mature nodules and the protective role of leghemoglobins as reactive oxygen species (ROS)/ reactive nitrogen species (RNS)-scavengers which in turn could inactivate nitrogenase. Following the same thematic, the original article of Berger et al. investigates the contribution of NR, as the main source of NO in plants, for the functioning of the symbiosis. In this paper, a dual role in nodule functioning is attributed to NR: generating NO as a signal for gene regulation and metabolic adaptation, and contributing to the energy supply under the hypoxic conditions prevailing inside the nodule.

Seed germination is another relevant issue for biologists and agriculture and the critical role of NO in breaking dormancy has been extensively demonstrated. In this Topic, Wang et al. analyze the crosstalk between NO and the inhibitory effects of ABA in releasing potato seed dormancy. Researchers probe in their original article the ABA-dependent decrease of the NO content and the NOS-like and NR activities in potato during sprouting. On the other hand, the promotion by NO of ABA catabolism and the inhibition of ABA biosynthesis ultimately induced dormancy release and tuber sprouting. In the same line, Ciacka et al. summarize in their mini-review the current knowledge on NO and other RNS contribution in the modulation of crucial events related to the preservation of seed vigor and/or regulation of seed longevity during aging. Authors highlight the interest of ROS-RNS cross-talk as NO counteracts ROS generation and stimulates the antioxidant system. Additionally, the authors indicate a concentration- and time-dependent effect of NO on ethylene, polyamines and ABA biosynthesis as well as the implication of the protein modification by *S*-nitrosylation in the regulation of the deterioration processes in seeds. Another kind of NO-derived modification, consistent with the nitration of unsaturated fatty acids, has been highlighted in the last few years in plant systems. This interaction results in the formation of nitro-fatty acids (NO_2_-FAs). In this regard, the original article of Mata-Pérez et al. reports the ability of nitro-linolenic acid (NO_2_-Ln) to move from roots to leaves in Arabidopsis plants. Moreover, given the potential of NO_2_-Ln to release NO at physiological pH and temperature, it can modulate the *in vitro* and *in vivo* levels of GSNO, the major mobile biological reservoir of NO bioactivity. The manuscript provided by Nabi et al. analyzes the interaction of NO with biological molecules too. In particular, they contribute to this Topic with an original sequencing study aiming at the functional characterization of NO-responsive domains of unknown function (DUF)-containing genes in Arabidopsis leaves. They identified 231 upregulated and 206 down-regulated DUF genes. The study focuses on *AtDUF569* given its significant increase in expression and interesting interactions with other proteins. This gene negatively regulates biotic stress responses and differentially regulates plant shoot and root growth under nitro-oxidative stress conditions.

In the context of the implication of NO in the tolerance against abiotic stress situations, Rather et al. highlights in their review the major aspects of copper (Cu)-induced toxicity in plants and summarize two possible strategies for NO to mitigate damages: the upregulation of the enzymatic and non-enzymatic antioxidant systems or defense genes, and the NO-participation in the root exclusion and/or activation of metal-chelating ligands such as metallothioneins and phytochelatins. In the same scope of environmental injuries, Pissolato et al. evidenced in their original article an interesting strategy for alleviating the negative effects of water deficit on sugarcane plants. These researchers demonstrate that the increase in the NO_3_ supply enhanced NO synthesis through NR which improves sugarcane performance under drought. Another environmental disaster is global warming and ozone layer depletion. Among others, nitrous oxide (N_2_O) is a potent greenhouse gas. The review of Timilsina et al. describes the mitochondrial reduction of the NR-derived NO to N_2_O under low oxygen conditions and proposes this route as a way to protect the mitochondrial and cellular integrity from the toxicity of NO accumulation under hypoxia and anoxia.

In summary, the variety of the work reported here (Figure 1) reinforces the already known relevance of NO in the biology of plant systems and highlights the complexity of its signaling roles. We hope that this Topic will encourage researchers to continue and initiate new studies in this exciting area where a plethora of molecular mechanisms remain unexplored.

**Figure 1:**
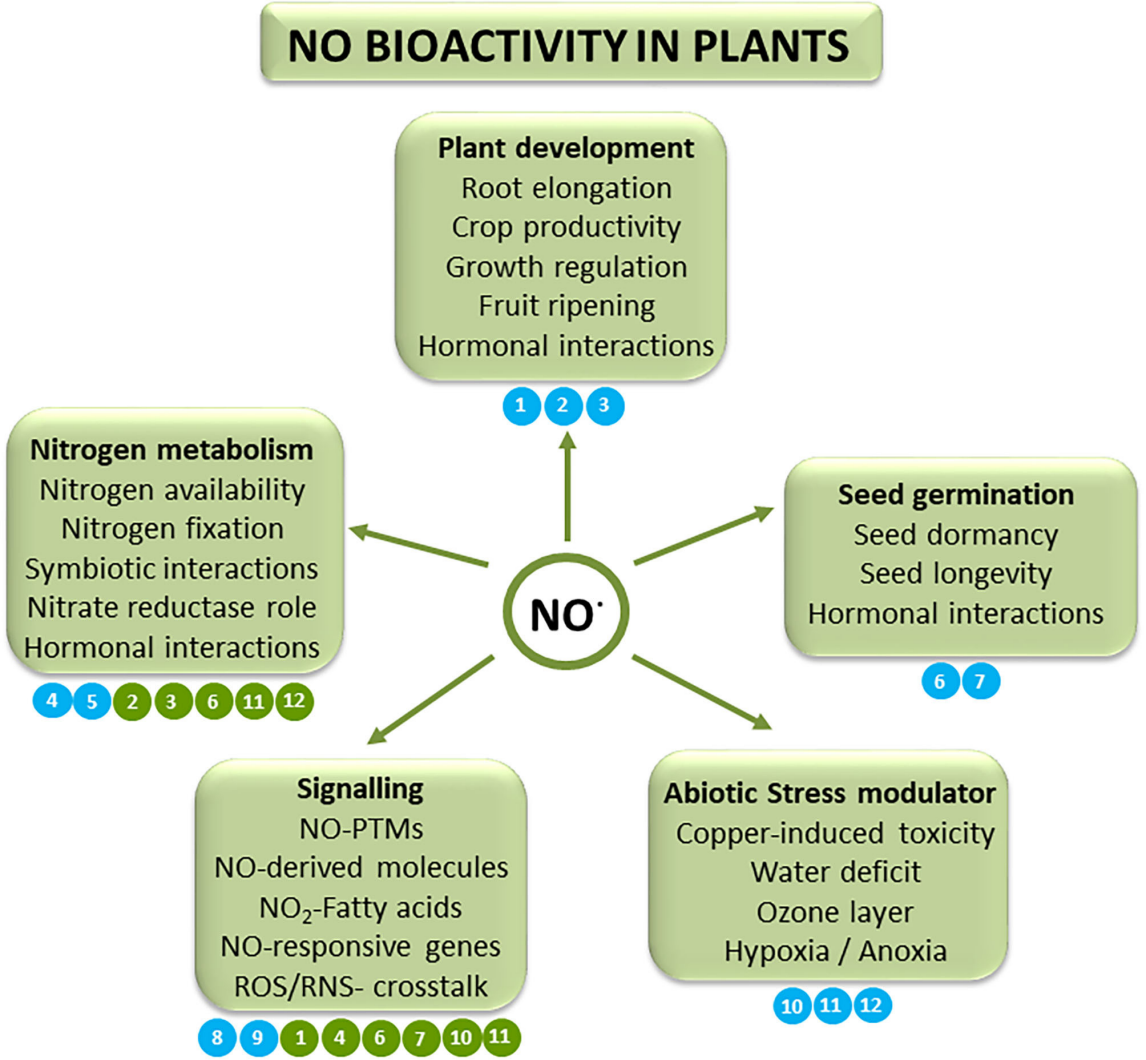
Schematic model of the NO bioactivity in plants in the scope of plant development, seed germination, abiotic stress situations, signaling, and nitrogen metabolism. The numbers in blue indicate the main subject matter covered in each article in the Research Topic. Additionally, the numbers in green indicate the relationship to additional topics. [1] Oláh et al. [2] Nejamkin et al. [3] Li et al. [4] Signorelli et al. [5] Berger et al. [6] Wang et al. [7] Ciacka et al. [8] Mata-Pérez et al. [9] Nabi et al. [10] Rather et al. [11] Pissolato et al. [12] Timilsina et al..

## Author Contributions

All authors listed have made a substantial, direct and intellectual contribution to the work, and approved it for publication.

## Conflict of Interest

The authors declare that the research was conducted in the absence of any commercial or financial relationships that could be construed as a potential conflict of interest.

